# Antimicrobial Prescribing during Infant Hospital Admissions in a Birth Cohort in Dhaka, Bangladesh

**DOI:** 10.1093/tropej/fmaa093

**Published:** 2020-11-22

**Authors:** Katherine Boone, Shaun K Morris, Sejal Doshi, Jason Black, Minhazul Mohsin, Tahmeed Ahmed, Abdullah Al Mahmud, Daniel E Roth

**Affiliations:** 1 Department of Paediatrics, The Hospital for Sick Children and University of Toronto, Toronto, ON M5G 1X8, Canada; 2 Centre for Global Child Health, The Hospital for Sick Children, Toronto, ON M5G 1X8, Canada; 3 Division of Infectious Diseases, The Hospital for Sick Children, Toronto, ON M5G 1X8, Canada; 4 Nutrition and Clinical Services Division, icddr,b, Dhaka 1212, Bangladesh

**Keywords:** low- and middle-income countries, Bangladesh, infants, inpatient, antimicrobial resistance, infectious diseases

## Abstract

Empirical antimicrobial use is common in hospitalized infants and may contribute to antimicrobial resistance in low- and middle-income countries. In this observational birth cohort study nested in a randomized controlled trial in Dhaka, Bangladesh, inpatient antimicrobial prescription data were extracted from serious adverse event forms completed for hospitalizations of infants (0–12 months of age). The primary outcome was the proportion of inpatient admissions where systemic antimicrobials were prescribed. Infant and hospitalization-related factors associated with antimicrobial prescriptions were determined. Among 1254 infants, there were 448 admissions to 32 facilities from 2014 to 2016. Antimicrobials were prescribed in 73% of admissions with a mean antimicrobial exposure rate of 0.25 antimicrobials per day of admission [95% confidence intervals (95% CIs): 0.24–0.27]. The most common antibiotics were aminoglycosides (29%), penicillins (26%) and third-generation cephalosporins (25%). In all, 58% of antibiotics were classified as ‘access’, 38% ‘watch’ and 1% ‘reserve’ using the World Health Organization (WHO) Essential Medicines List classification. WHO-recommended antimicrobial regimens were used in 68% of neonatal sepsis and 9% of lower respiratory tract infection (LRTI) admissions. ‘Watch’ antimicrobials were used in 26% of neonatal sepsis and 76% of LRTI admissions. Compared with private facilities, antimicrobial prescription rates were lower at government [rate ratio (RR) 0.71; 95% CI: 0.61–0.83] and charitable facilities (RR 0.39; 95% CI: 0.28–0.53), after adjustment for household wealth index and parental education. Younger infant age, older maternal age and longer admission were associated with higher prescription rates. These findings highlight the need for paediatric antimicrobial stewardship programs in Bangladesh.

## INTRODUCTION

Antimicrobial resistance (AMR) is a global health problem of particular concern in low- and middle-income countries (LMICs) where there is a high burden of infectious diseases, second-line agents may be less accessible, drugs may be obtained without physician prescription, and antimicrobial stewardship programs (AMSPs) are uncommon [1–5]. Prescribing patterns that contribute to AMR include administration of antibiotics for non-bacterial illness, unnecessary or excessive use of broad-spectrum antibiotics and inappropriate antibiotic choice or duration of treatment for an identified pathogen [4, 5]. Concern about AMR is particularly relevant in paediatrics considering how frequently children are prescribed antimicrobials, even in high-income settings [6]. Moreover, guidelines recommend broad-spectrum antibiotics as initial empiric treatment for young infants with suspected sepsis or non-specific signs of illness [7], although causative bacterial pathogens are rarely isolated [8]. Antibiotic use incurs a risk of drug-related adverse events and treatment failure, particularly if dosage, activity spectrum or duration is inappropriate [9, 10]. Additionally, antibiotics may reduce intestinal microbiome diversity, which is particularly relevant in infancy [11].

Several studies have described emerging AMR of common and clinically important bacteria in Bangladesh [12–16]. Outpatient antimicrobial overuse in Bangladesh has been reported and is attributable to both self-administration and physician prescriptions [15, 17, 18]. Cephalosporins are commonly used for mild symptoms [18, 19], and macrolides are often administered for acute non-bloody diarrhoea [17]. A recent study of antimicrobial use in longitudinal birth cohorts in eight LMICs found that children in Dhaka, Bangladesh received on average 10.3 antimicrobial courses per child-year up to 2 years of age compared with the global cohort average of 4.9 antimicrobial courses per child-year, and >98% of infants in Dhaka had received antibiotics by 6 months of age [20]. In comparison, children younger than 2 years in Europe and the USA reportedly have <2 prescribed courses of antibiotics per child-year [21]. Some studies have addressed inpatient paediatric antimicrobial prescribing in Bangladesh [15, 17, 22–24], but none has focused on infants, a population who are frequently admitted for parenteral antibiotics.

Leveraging data collected during a vitamin D supplementation trial, we aimed to characterize the use of antibiotics among infants hospitalized in Dhaka and to identify patient and facility level factors associated with antimicrobial exposure. The overall goal of the research is to explore antimicrobial prescribing patterns that may increase the risk of AMR in this population and highlight the potential benefit of AMSPs to promote appropriate antimicrobial use in paediatric practice.

## METHODS

This study was a *post hoc* analysis of antimicrobial prescription and clinical data recorded for the sub-group of participants in the Maternal Vitamin D for Infant Growth (MDIG) trial who were admitted to hospital in the first 12 months of life (clinicaltrials.gov # NCT01924013). The MDIG trial was a randomized, placebo-controlled, dose-ranging trial of maternal vitamin D supplementation in Dhaka, Bangladesh in which 1300 pregnant women were enrolled from March 2014 to September 2015, and infant hospitalizations occurred from July 2014 to December 2016 [25]. Previous analyses did not show any effect of the vitamin D intervention on the risk of infant hospitalization [25].

For each hospitalization, research physicians collected information from the patient’s medical chart and completed a serious adverse event (SAE) form. Data collected included facility type (classified *post hoc* as private, government or charitable), admission/discharge dates and diagnoses, presenting signs/symptoms and inpatient medications (including prescribed antimicrobials). Antimicrobial durations and dosages were usually not recorded in the SAE form and therefore were not analysed. Typically, patients pay out-of-pocket for care at private facilities, whereas government facilities funded by the Bangladesh government and charitable facilities run by non-governmental organizations provide care at no or low cost to patients. For infants enrolled in the MDIG trial, hospital costs were usually covered by the study regardless of facility type. For each admission, facility type was based on the hospital to which the infant was first admitted. All infants were enrolled in Dhaka, and the majority of admissions was at facilities within Dhaka; only 3 (0.6%) admissions were at facilities located outside of the city. Data were not collected about AMSPs at the admitting hospitals.

Antibiotic exposure was described as the number/proportion of hospital admissions during which at least one antimicrobial was prescribed and the antimicrobial prescription rate, expressed as the average number of distinct antimicrobials prescribed per patient-day of hospitalization, which reflects the range of distinct antimicrobials to which infants were exposed but does not take into account the number of doses or days of administration of each drug. Antimicrobials were stratified by class and using the World Health Organization (WHO) 2017 Essential Medicines List (EML) classification [26] (see Supplementary Table S1): ‘access’ if they should be widely available, affordable and quality-assured (e.g., ampicillin, cefazolin); ‘watch’ if they have a higher resistance potential and should be limited to a specific, limited number of indications (e.g., third-generation cephalosporins, carbapenems) and ‘reserve’ if they should be used as a last resort in highly specific patients to preserve effectiveness (e.g., fourth-generation cephalosporins, polymyxins) [27]. Using the WHO EML classification, we calculated an ‘access-to-watch’ ratio [28]. Oral or intravenous antifungal, anti-parasitic and antiviral drugs were included but were prescribed rarely or not at all. Topical antimicrobials were excluded.

Associations between antibiotic prescription rate and the following factors were estimated: infant age, gestational age at birth, small for gestational age (defined as weight for gestational age z-score below the 10th percentile, based on the Intergrowth 21st Neonatal Standards), low birth weight (defined as weight <2500 g at birth), duration of admission, household wealth index [25], parental education level and employment, maternal parity and age and facility type (private, government or charitable). Infant age was categorized as 0–28 days (neonatal), 29–90 days (1–3 months) and 91–365 days (3–12 months).

Rate ratios (RRs) with 95% confidence intervals (95% CIs) were estimated using Poisson regression, whereby each admission was the unit of analysis, the count of different antibiotics per admission was the outcome, log-transformed days of admission was the offset and the non-independence of an infant’s repeat admissions was addressed using generalized estimating equations and robust standard errors. We could not make causal inferences about determinants of antimicrobial prescribing; rather, we considered unadjusted RRs as a means of identifying subgroups of infants who may be at lower/higher risk of antimicrobial exposure. However, due to the observed unadjusted association between antimicrobial prescription rates and facility type, a *post hoc* multivariable analysis of facility type was conducted in which we adjusted for wealth index and maternal/paternal education level and stratified by age group. In further analyses, antimicrobial exposure was assessed by facility type or for selected primary admitting diagnoses [suspected neonatal sepsis, acute lower respiratory tract infections (LRTIs)] for which empirical antibiotics are widely used and prescription patterns could be compared with WHO guidelines [29]. The category of acute LRTIs included pneumonia and bronchiolitis, which were not readily distinguished based on medical records. We also considered antibiotic exposure for admitting diagnoses for which antibiotics are not usually indicated (acute gastroenteritis, neonatal jaundice). Given the limited detail in SAE reports, these admissions could only be assigned a primary diagnosis. For some infants with a primary admitting diagnosis of gastroenteritis or jaundice, antimicrobials may have been prescribed for another indication but we assumed this would be relatively infrequent.

Analyses were performed using SAS (version 9.4). Participants provided written informed consent for participation in the trial. The original trial protocol and a separate protocol for use of de-identified data for this sub-study were approved by research ethics boards at the Hospital for Sick Children (Canada) and icddr,b (Bangladesh).

## RESULTS

**Fig. 1.**
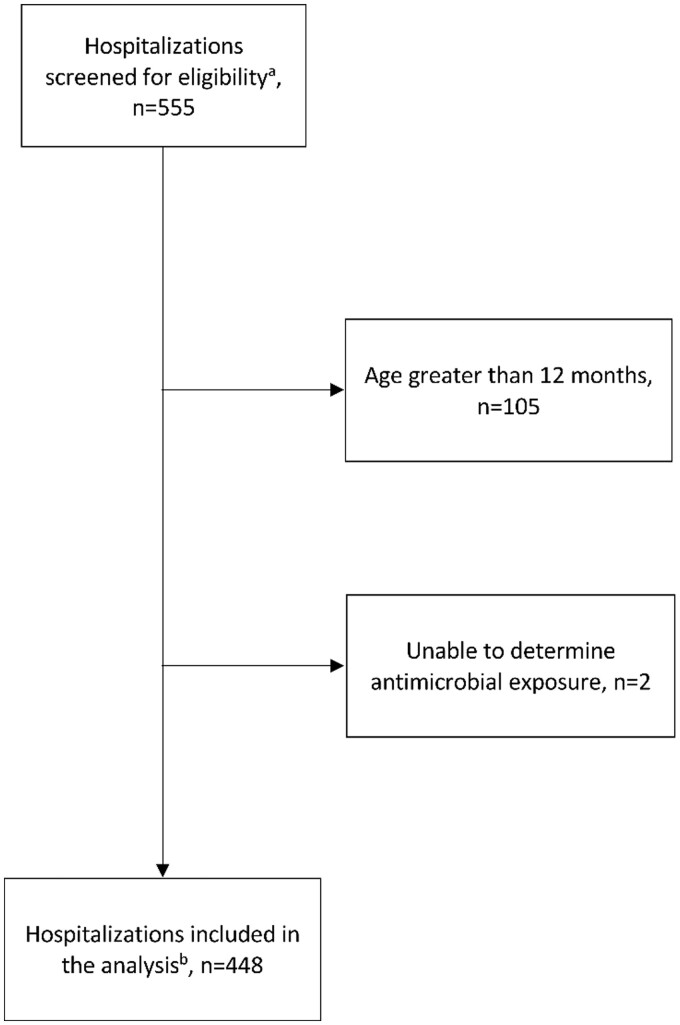
Inclusion criteria for patients enrolled in the trial. ^a^All admissions for children enrolled in the MDIG trial. ^b^Ages 0–12 months at the time of admission, between July 2014 and December 2016

Of 555 paediatric admissions in the MDIG database, 105 were excluded due to age >12 months at time of admission, and 2 were excluded due to inability to determine antibiotic exposure (Fig. 1). Therefore, the sample size was 448 admissions at 32 different facilities in Dhaka, Bangladesh involving 373 infants, of whom 314 were admitted once, 48 twice, 7 three times, 3 four times and 1 five times in the first year of life. Of the 448 admissions, 31 involved at least one transfer to another facility (6.9%), of which 22 involved a transfer to a different facility type (4.9%). The most common transfer was from a government to a private facility (19/313 admissions to government facilities, 6.1%). The median length of stay was 5 days (IQR 5). There were 16 (3.6%) infant deaths in hospital, most commonly in the neonatal period (88%, *n* = 14). Most infants were admitted to government facilities (70%; *n* = 313) and less to private (17%; *n* = 78) and charitable (13%; *n* = 57) facilities. Common admitting diagnoses are listed in Table 1. Overall, 88% of SAE reports were completed within 1 week of discharge (median: 1 day; IQR: 0–4 days).

**Table 1:** Common admitting diagnoses in a birth cohort of infants in Dhaka, Bangladesh (N = 448), overall and by age group

Admitting diagnosis^a^	All infants, *n* (%)^b^	≤28 days, *n* (%)^c^	29 days to 3 months, *n* (%)^c^	>3–12 months, *n* (%)^c^
Total admissions	448	195 (44)^b^	39 (8.7)^b^	214 (48)^b^
Diarrhoea/gastroenteritis (non-bloody)	102 (23)	4 (2.1)	10 (26)	88 (41)
Hyperbilirubinaemia/jaundice	43 (9.6)	43 (22)	0	0
Lower respiratory tract infection (includes pneumonia, bronchiolitis)	124 (28)	7 (3.6)	23 (59)	94 (44)
Meconium aspiration syndrome	26 (5.8)	26 (13)	0	0
Perinatal asphyxia/hypoxic–ischaemic encephalopathy	23 (5.1)	23 (12)	0	0
Sepsis/serious bacterial infection (includes meningitis)^d^	36 (8)	31 (16)	1 (2.6)	4 (1.9)

a Diagnoses assigned by study physician based on review of medical record and/or communication with treating medical staff.

b Percentage represents the proportion of total admissions including all age groups.

c Percentage represents the proportion of all admissions within the specified age group (with the exception of the first row).

d Includes clinically suspected sepsis or serious bacterial infection.

**Fig. 2.**
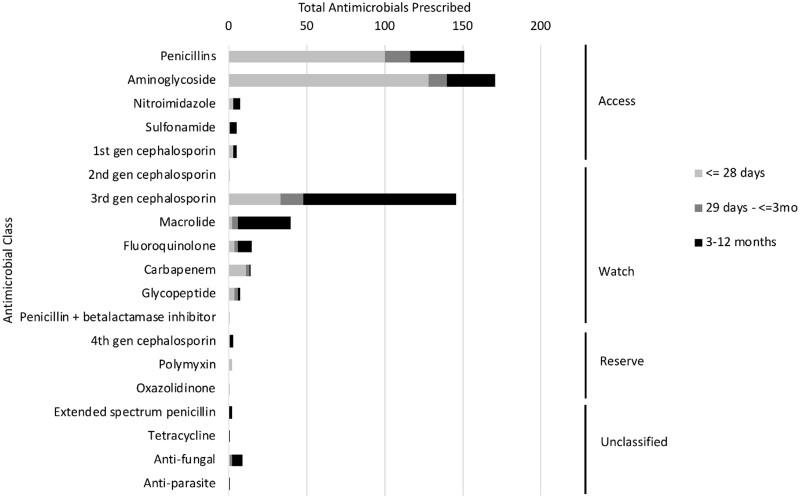
Total antimicrobials prescribed by antimicrobial class, WHO Essential Medicines List (EML) classification,^a^ and age at admission in a birth cohort in Dhaka, Bangladesh (*N* = 582 antimicrobial prescriptions). ^a^WHO EML Classification: antibiotics are classified as ‘access’ if they should be widely available, affordable and quality-assured; ‘watch’ if they have a higher resistance potential and should be used for a specific, limited number of indications or ‘reserve’ if they should be used as a last resort in highly specific patients and settings, to preserve effectiveness. There were 13 prescriptions for unclassified antimicrobials.

In 73% (*n* = 329) of admissions, at least one systemic antimicrobial was prescribed; the overall antimicrobial prescription rate was 0.25 antimicrobials per infant per day of hospitalization (95% CI: 0.24–0.27), with some variation across groups defined by age and primary admitting diagnosis (Supplementary Table S2). Aminoglycosides (29% of all prescriptions), penicillins (26%) and third-generation cephalosporins (25%) were most prescribed. By age group, the most prescribed antimicrobials were aminoglycosides (43% of antimicrobials, 128/296) for neonates, penicillins (30%, 16/54) for infants 1–3 months and third-generation cephalosporins (42%, 98/232) for infants 3–12 months (Fig. 2). A total of 582 antimicrobials were prescribed of which 58% (*n* = 339) were ‘access’, 38% (*n* = 224) ‘watch’ and 1% (*n* = 6) ‘reserve’ (Fig. 2). Across all age groups, the most common ‘access’ antimicrobials were aminoglycosides and penicillins (Fig. 2). Overall, the most common ‘watch’ antimicrobials were third-generation cephalosporins and macrolides, but carbapenems were more common than macrolides in neonates (Fig. 2).

**Fig. 3.**
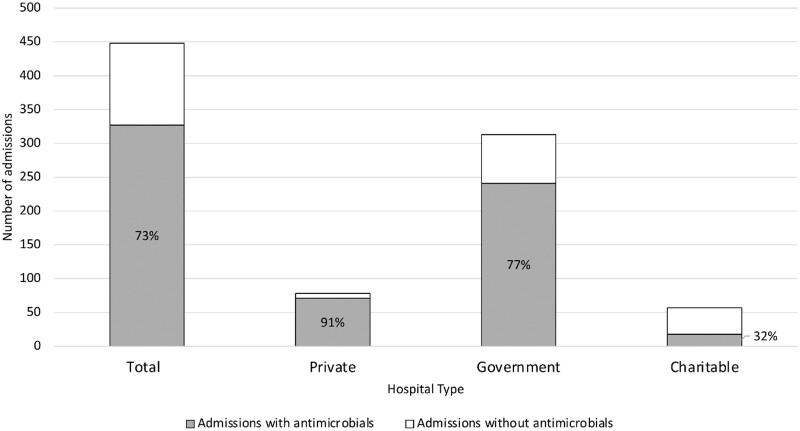
Number of admissions in which at least one antimicrobial was prescribed, overall and by facility type,^a^ for children younger than 1 year of age in a birth cohort in Dhaka, Bangladesh (*N* = 448 admissions). ^a^ Facilities were classified as private if care is paid for by patients/families, government if care is provided by the Bangladesh government and charitable if care is provided by non-governmental organizations at no or very low cost to patients.

**Fig. 4.**
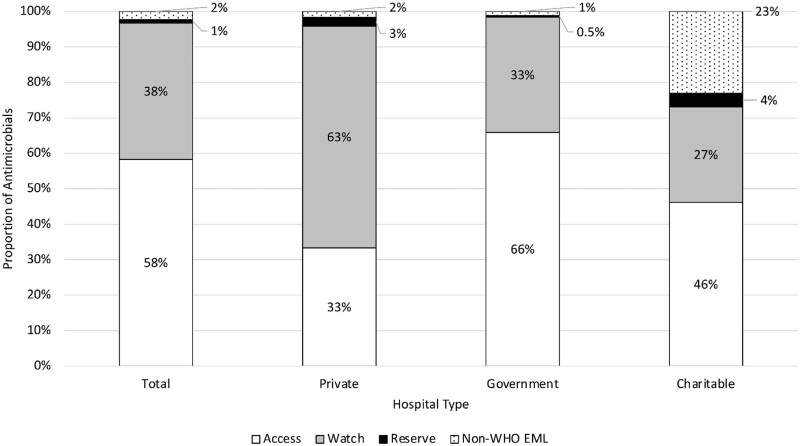
Percentage^a^ of each WHO Essential Medicines List (EML) antimicrobial type prescribed, overall and by facility type,^b^ for children younger than 1 year of age in a birth cohort in Dhaka, Bangladesh (*N* = 582 antimicrobials overall; *N* = 120, 436 and 26 at private, government and charitable facilities, respectively). ^a^Percentage of the total antimicrobials prescribed. ^b^ Facilities were classified as private if care is paid for by patients/families, government if care is provided by the Bangladesh government and charitable if care is provided by non-governmental organizations at no or very low cost to patients.

Antimicrobials were most prescribed during admissions for which the initial admitting hospital was a private facility and least at charitable facilities (Fig. 3). The ‘access-to-watch’ ratio was 1.51 overall, but varied across facility type (0.53, 1.71 and 2.02 for private, charitable and government facilities, respectively). ‘Access’ antimicrobials were the most prescribed antimicrobial type at government (66% of all antimicrobials prescribed, 287/436) and charitable facilities (46%, 12/26), but ‘watch’ antimicrobials were the most prescribed type at private facilities (63%, 75/120) (Fig. 4). The most common non-WHO EML antimicrobial (unclassified) among all facility types was nystatin (oral antifungal; 9/13 unclassified prescriptions). Unclassified antimicrobials (antifungals, tetracyclines and anti-parasitic drugs) were most prescribed at charitable facilities. However, the majority of unclassified antimicrobials prescribed at charitable facilities (5/6 prescriptions) was nystatin, all of which were prescribed at one facility.

Several factors were associated with lower antimicrobial prescription rates including older age (3–12 months), longer duration of admission and higher maternal age (Table 2). Admission to government or charitable facilities was associated with a lower rate compared with private facilities, even after adjusting for wealth index and maternal/paternal education (Table 3).

**Table 2:** Association of patient characteristics with antimicrobial prescription rate among infants hospitalized in Dhaka, Bangladesh (N = 448)

Patient characteristic	Admissions, *n* (%)	At least one antimicrobial prescribed, *n* (%^a^)	Antimicrobial prescription rate (drugs per infant per day)	Unadjusted rate ratio (95% CI)	*p*
Overall	448 (100)	329 (73)	0.25 (0.24–0.27)	—	—
Sex					
Female	251 (56)	187 (75)	0.27 (0.24–0.29)	Ref	
Male	197 (44)	142 (72)	0.24 (0.19–0.30)	0.89 (0.77–1.04)	0.14
Age					
0 to ≤28 days	195 (44)	134 (69)	0.27 (0.25–0.31)	Ref	
29 days to 3 months of age	39 (9)	32 (82)	0.25 (0.18–0.36)	0.93 (0.73–1.17)	0.52
3–12 months of age	214 (48)	163 (76)	0.23 (0.18–0.30)	0.85 (0.73–0.99)	0.03
Preterm birth					
No	387 (86)	285 (74)	0.26 (0.20–0.33)	Ref	
Yes	61 (14)	44 (72)	0.25 (0.16–0.41)	0.98 (0.76–1.25)	0.86
Low birth weight^b^					
No	193 (73)	136 (70)	0.24 (0.21–0.27)	Ref	
Yes	70 (27)	44 (63)	0.21 (0.15–0.31)	0.89 (0.69–1.15)	0.38
Small for gestational age^b^					
No	136 (52)	96 (71)	0.24 (0.21–0.28)	Ref	
Yes	127 (48)	84 (66)	0.22 (0.16–0.31)	0.92 (0.75–1.14)	0.46
Duration of admission					
Below median (5 days)	220 (49)	121 (55)	0.38 (0.33–0.43)	Ref	
At or above median (5 days)	228 (51)	208 (91)	0.22 (0.17–0.29)	0.58 (0.50–0.68)	<0.001
Household wealth index					
Below median (0.035)	225 (50)	170 (76)	0.25 (0.23–0.28)	Ref	
At or above median (0.035)	223 (50)	159 (71)	0.26 (0.20–0.33)	1.03 (0.89–1.20)	0.65
Maternal education level					
No schooling	21 (5)	14 (67)	0.21 (0.16–0.28)	Ref	
Primary incomplete	100 (22)	74 (74)	0.24 (0.13–0.44)	1.14 (0.83–1.57)	0.95
Primary complete	71 (16)	50 (70)	0.22 (0.12–0.40)	1.01 (0.72–1.42)	0.41
Secondary incomplete	156 (35)	113 (72)	0.27 (0.15–0.47)	1.24 (0.92–1.69)	0.16
Secondary complete	100 (22)	78 (78)	0.28 (0.16–0.50)	1.33 (0.99–1.79)	0.06
Maternal employment^c^					
Homemaker	410 (92)	297 (72)	0.25 (0.23–0.27)	Ref	
Employed	36 (8)	30 (83)	0.28 (0.22–0.36)	1.12 (0.93–1.34)	0.23
Maternal age					
Below median (22 years)	189 (42)	143 (76)	0.28 (0.25–0.31)	Ref	
At or above median (22 years)	259 (58)	186 (72)	0.24 (0.19–0.30)	0.86 (0.74–0.99)	0.04
Paternal education					
No schooling	23 (5)	17 (74)	0.19 (0.14–0.24)	Ref	
Primary incomplete	69 (15)	52 (75)	0.26 (0.14–0.47)	1.38 (0.87–2.04)	0.15
Primary complete	58 (13)	44 (76)	0.29 (0.16–0.53)	1.57 (1.01–2.44)	0.04
Secondary incomplete	156 (35)	115 (74)	0.26 (0.14–0.46)	1.39 (0.92–2.08)	0.12
Secondary complete	101 (23)	73 (72)	0.25 (0.14–0.44)	1.34 (0.87–2.04)	0.18
Unknown/missing	41 (9)	28 (68)	0.23 (0.12–0.46)	1.26 (0.77–2.05)	0.35
Paternal employment^d^					
Jobless	15 (3)	12 (80)	0.27 (0.20–0.37)	Ref	
Day labourer or rickshaw driver	60 (13)	39 (65)	0.20 (0.10–0.40)	0.73 (0.50–1.06)	0.10
Private business owner or professional	139 (31)	96 (69)	0.26 (0.13–0.49)	0.94 (0.67–1.32)	0.72
Salaried job	214 (48)	168 (79)	0.27 (0.14–0.51)	0.98 (0.71–1.36)	0.91
Other	17 (4)	12 (71)	0.23 (0.10–0.54)	0.84 (0.49–1.44)	0.52

a Percent of admissions within the listed subgroup (row).

b Data were missing for 185 admissions.

c Data were missing for two admissions.

d Data were missing for three admissions.

Preterm birth: gestational age <37 weeks at birth.

Low birth weight: weight <2500 g at birth.

Small for gestational age: weight for gestational age *z*-score below the 10th percentile based on the Intergrowth 21st Neonatal Standards.

Household wealth index: higher scores indicate greater household asset ownership relative to other participants of the MDIG trial (25).

**Table 3:** Association of facility type with antimicrobial prescription rate, overall and by age group (0–12 months) among infants in Dhaka, Bangladesh (N = 448 admissions)

Facility type	All infants				0–28 days		29 days to 3 months		>3–12 months	
	Unadjusted rate ratio (95% CI)	*p*	Adjusted rate ratio (95% CI)^a^	*p*	Adjusted rate ratio (95% CI)^a^	*p*	Adjusted rate ratio (95% CI)^a^	*p*	Adjusted rate ratio (95% CI)^a^	*p*
Private	Ref		Ref		Ref		Ref		Ref	
Government	0.70 (0.60–0.82)	0.001	0.71 (0.61–0.83)	0.001	0.78 (0.45–1.3)	0.35	0.78 (0.55–1.1)	0.14	0.57 (0.46–0.70)	<0.0001
Charitable	0.39 (0.29–0.53)	0.001	0.39 (0.28–0.53)	0.001	0.25 (0.14–0.45)	<0.0001	0.43 (0.25–0.76)	0.003	0.43 (0.29–0.62)	<0.0001

a Adjusted for household wealth index and parental education.

b Facilities were classified as private if care is paid for by patients/families, government if care is provided by the Bangladesh government, and charitable if care is provided by non-governmental organizations at no or very low cost to patients. For admissions in which infants were transferred between facilities, facility type was based on the first hospital to which the infant was admitted.

Antimicrobials were used in 51% (52/102) of gastroenteritis and 28% (12/43) of neonatal jaundice admissions. For suspected neonatal sepsis (*n* = 31), a WHO-recommended regimen [ampicillin and gentamicin (*n* = 20) or cloxacillin and gentamicin (*n* = 1)] was used in 68% of admissions, whereas ‘watch’ antimicrobials were used in 26% of cases (*n* = 8) (amikacin, ceftazidime, ceftriaxone, meropenem and vancomycin) and ‘reserve’ in 6% (*n* = 2) (cefepime and colistimethate). Antibiotics were prescribed in 98% of 124 LRTI admissions. The WHO-recommended regimen for severe pneumonia (ampicillin and gentamicin) was used in 9% of admissions (*n* = 11), ampicillin was prescribed alone in three admissions and ‘watch’ antimicrobials (primarily ceftriaxone) were used in 76% of LRTI admissions (*n* = 94); a ‘reserve’ antimicrobial (cefepime) was used in two LRTI admissions.

## DISCUSSION

At least one antimicrobial was prescribed in a majority (73%) of 448 hospitalizations in a cohort of 373 infants in Dhaka, Bangladesh from 2014 to 2016. Neonates had the highest antimicrobial prescription rate, in part because multiple drugs were frequently co-administered. The observation of frequent antibiotic use in this sample of paediatric inpatients in Dhaka, Bangladesh was consistent with prior studies in Bangladesh [17, 22–24] and other LMICs [30], including recent studies that have highlighted the relatively high antimicrobial exposure of hospitalized newborns and infants in India [31], South Africa [32], Ethiopia [33, 34] and Ghana [35].

More than one-third of antimicrobials prescribed to infants in this study were ‘watch’ agents, indicating a higher resistance potential [27]. The frequent prescription of second-line antimicrobials was reflected in the low ‘access-to-watch’ ratio of ∼1.5 overall (e.g., 3 ‘access’ for every 2 ‘watch’ prescriptions). Among 70 middle- and high-income countries in a recent study of sales of oral antibiotics prescribed to young children, Bangladesh had the lowest ‘access-to-watch’ ratio (0.5, compared with the overall average of 6) [28]. Despite the frequent use of ‘watch’ antibiotics, we documented relatively infrequent prescription of ‘reserve’ antibiotics, consistent with paediatric prescribing patterns observed in other studies [36].

Infants often received antimicrobials when the admitting diagnosis was likely a viral illness or a condition for which antibiotics do not usually confer benefit (e.g., gastroenteritis, jaundice). Ahmed *et al*. [17] previously reported in a single-centre study in a rural hospital near Dhaka that 80% of children admitted with diarrhoea received antibiotics, which the authors deemed inappropriate in most cases. In the present study, antimicrobials were prescribed in 98% of LRTI admissions, a condition for which empiric antibiotic therapy is recommended in children who meet WHO clinical criteria for pneumonia [29]; however, we found that ‘watch’ antimicrobials, such as third-generation cephalosporins, were more commonly used than the WHO-recommended first-line regimen [26]. Similarly, Rashid *et al.* [23] found that ceftriaxone was the most common treatment for children with severe or very severe pneumonia at a private paediatric hospital in Dhaka, and cephalosporins were often used for outpatient treatment of acute respiratory infections in young children in Dhaka [20]. These patterns are not unique to Bangladesh, as ceftriaxone (a ‘watch’ antibiotic) is one of the most widely used antibiotics in inpatient paediatrics [36, 37]. A recent study of paediatric inpatient antimicrobial exposure in 56 countries (not including Bangladesh) found that ‘watch’ antibiotics were used in a majority of LRTI cases in most countries including high-income countries [36]. ‘Access’ antibiotics were more often selected for neonatal sepsis compared with LRTI, yet there was substantial variability among surveyed institutions and usage ranged from 35% to 56% across world regions [36]. WHO-recommended regimens for neonatal sepsis were frequently selected in the present cohort (68%).

In Bangladesh, common childhood illnesses are frequently treated with oral and injectable antibiotics in the outpatient setting prior to hospital admission [17, 23], which may lead admitting physicians to escalate therapy to second-line agents for a perceived failure of response to outpatient therapy. Furthermore, AMR is remarkably common in Bangladesh, including very high rates of beta-lactamase resistant Enterobacteriaceae [38]; awareness of AMR may contribute to physician preferences for watch/reserve antibiotics. A recent survey of neonatal units in LMICs found that all three of the surveyed units in Bangladesh listed ‘watch’ antibiotics as options for empirical treatment of early- and late-onset sepsis [39]. Use of carbapenems or other watch/reserve drugs may be increasingly used by physicians in South Asia since a minority of bacterial isolates causing neonatal sepsis have been estimated to be susceptible to WHO-recommended first- and second-line antibiotics in India and Pakistan [40].

A key finding of this study was that inpatient antibiotic prescription practices varied by initial admitting facility type, even after adjusting for family characteristics that might have influenced the type of facility to which an infant was admitted. Infants initially admitted to private facilities were significantly more likely to receive antimicrobials than those initially admitted to government and charitable facilities. Moreover, the ‘access-to-watch’ ratio of 0.53 at private facilities was substantially lower than at government and charitable facilities (2.02 and 1.71, respectively). The discrepancy in overall prescription rates and types of antibiotics between private and government/charitable facilities may be related to the acuity of illness, given that the sickest infants were likely to be transferred to private facilities for more advanced care. In addition, in this study costs of hospitalizations including drugs were usually covered by the study, which may have inadvertently increased physicians’ propensity to forego first-line agents or advance more quickly to second-line agents. A recent study in China found that paediatric antimicrobial prescribing is more frequent and more likely to be inappropriate at ‘general’ and ‘non-tertiary’ hospitals compared with tertiary-care children’s hospitals [41]. However, most prior studies of paediatric inpatient antimicrobial prescribing in LMICs have been conducted in teaching hospitals [30] and therefore may underestimate the extent of inappropriate antimicrobial use in other facility types.

The strength of this study was the passive observation of antibiotic prescription patterns during admissions at 32 different facilities, whereas other recent paediatric inpatient studies in Bangladesh were based on single-centre studies [17, 23, 24]. Inpatient medical record keeping practices are variable and rarely electronic in Bangladesh, such that antimicrobial prescribing databases are unlikely to be maintained. In this study, data collection by research personnel, rather than by health care providers involved in patient care, revealed practice patterns in the absence of interventions designed to modify antimicrobial prescribing. Additionally, the data were conformable to the WHO EML classification and could be used as a basis for comparison to other studies.

A limitation of this study was that specific dosages and durations of antimicrobials were unavailable but may have enabled analyses of over- or under-treatment, adverse effects and propensity to generate AMR. We were unable to evaluate the appropriateness of antimicrobial prescriptions at the individual patient/admission level due to the absence of detailed clinical information; primary diagnosis may have been an inadequate classifier if antimicrobials were indicated for a reason other than a viral or non-infectious admitting diagnosis. Blood cultures are not routinely performed in many hospitals in Bangladesh, but it remains possible that for some hospitalizations, culture results not recorded in the SAE reports may have influenced clinicians’ decisions to use a treatment regimen that did not conform to empiric treatment guidelines. In the LRTI group, we were unable to differentiate bronchiolitis (assumed to be viral) from pneumonia (usually bacterial), a distinction that influences antimicrobial prescribing. Outpatient prescriptions preceding hospitalization were not available; therefore, we could not determine if patients had failed outpatient management with a first-line (‘access’) oral drug, which may prompt physicians to prescribe a second-line agent as inpatient treatment. The classification of admission by facility type was based on the initial hospital to which the infant was admitted; because infants were occasionally transferred to private facilities for more advanced care, this approach may have attenuated observed differences in prescription patterns between facility types.

## Conclusions

This study demonstrated the high rate of antibiotic exposure of infants admitted to hospitals in Dhaka, Bangladesh. Antimicrobials were often ‘watch’ drugs, which is concerning due to their contribution to AMR. These findings underscore the need for AMSPs, the aim of which is to ensure access to appropriate antibiotics and adherence to treatment guidelines and for which successful implementation requires engagement at all levels of the health system [42]. AMSPs have been shown to curtail AMR in high-income countries and there is growing recognition of their importance in LMICs, particularly in paediatric populations [3, 43, 44]. The Bangladesh National Action Plan on Antimicrobial Resistance Containment was launched in 2017 [45] but does not specifically mention the critical role of AMSPs in promoting appropriate inpatient prescribing practices. However, building on emerging experience with AMSPs in Dhaka [24], strong consideration should be given to the development of paediatric AMSPs to rationalize inpatient prescription of antimicrobials to hospitalized infants in Bangladesh.

## SUPPLEMENTARY DATA

Supplementary data are available at *Journal of Tropical Pediatrics* online.

## Supplementary Material

fmaa093_Supplementary_Data
