# Supplementary material for: Antimicrobial Prescribing during Infant Hospital Admissions in a Birth Cohort in Dhaka, Bangladesh
Source: J Trop Pediatr. 2020 Nov 22;67(3):fmaa093. doi: 10.1093/tropej/fmaa093 (PMC8319631; doi:10.1093/tropej/fmaa093)
Supplement: fmaa093_Supplementary_Data [file fmaa093_supplementary_data.docx]

**Supplementary Table 1. Antimicrobial classifications according to the World Health Organization Essential Medicine List 2017**

| Antimicrobial name | Antimicrobial class | WHO EML classification |
| --- | --- | --- |
| Amoxicillin | Penicillins | Access |
| Ampicillin | Penicillins | Access |
| Cloxacillin | Penicillins | Access |
| Gentamicin | Aminoglycoside | Access |
| Amikacin | Aminoglycoside | Access |
| Netilmicin | Aminoglycoside | Access^a^ |
| Netromycin | Aminoglycoside | Access^a^ |
| Metronidazole | Nitroimidazole | Access |
| Trimethoprim / Sulfamethoxazole | Sulfonamide | Access |
| Cephalexin | 1st gen cephalosporin | Access |
| Cefradoxil | 1st gen cephalosporin | Access^a^ |
| Cefuroxime | 2nd gen cephalosporin | Watch^b^ |
| Ceftriaxone | 3rd gen cephalosporin | Watch |
| Cefotaxime | 3rd gen cephalosporin | Watch |
| Cefixime | 3rd gen cephalosporin | Watch |
| Cefpodoxime | 3rd gen cephalosporin | Watch |
| Erythromycin | Macrolide | Watch |
| Clarithromycin | Macrolide | Watch |
| Azithromycin | Macrolide | Watch |
| Ciprofloxacin | Fluoroquinolone | Watch |
| Meropenem | Carbapenem | Watch |
| Imipenem | Carbapenem | Watch |
| Vancomycin | Glycopeptide | Watch |
| Piperacillin / tazobactam | Penicillin + beta lactamase inhibitor | Watch |
| Cefepime | 4th gen cephalosporin | Reserve |
| Colistimethate | Polymyxin | Reserve |
| Linezolid | Oxazolidinone | Reserve |
| Pivmecillinam^c^ | Extended spectrum penicillin | Unclassified |
| Tetracycline | Tetracycline | Unclassified |
| Nystatin | Anti-fungal | Unclassified |
| Nitazoxanide | Anti-parasite | Unclassified |

^a^ Antibiotic not listed in the 2017 World Health Organization (WHO) Essential Medicines List (EML). Classification based on other antibiotics of the same class as per the 2017 EML.

^b^ Antibiotic not classified in the 2017 WHO EML; classification based on 2019 WHO EML.

^c^ This is an extended-spectrum penicillin antibiotic. This antibiotic was not listed in either the 2017 or 2019 WHO EML. Given that the resistance potential of this antimicrobial may be different from the penicillin group as a whole, it was not grouped with its respective class and was instead unclassified.

**Supplementary Table 2. Antimicrobial** **exposure among infants hospitalized in Dhaka, Bangladesh (N=448), stratified by age group and admitting diagnosis**

| Patient Characteristic | Admissions,  n (%) | At least one antimicrobial prescribed, n (%^a^) | Antimicrobial prescription rate (drugs per infant per day) |
| --- | --- | --- | --- |
| Diarrhea / gastroenteritis (non-bloody) | | | |
| 0 to <=28 days | 4 (3.9) | 2 (50) | 0.22 (0.09 to 0.52) |
| 29 days to 3 months of age | 10 (9.8) | 6 (60) | 0.27 (0.1 to 0.72) |
| 3 to 12 months of age | 88 (86) | 44 (50) | 0.26 (0.21 to 0.32) |
| Hyperbilirubinemia / jaundice |  |  |  |
| 0 to <=28 days | 43 (100) | 12 (28) | 0.13 (0.08 to 0.21) |
| 29 days to 3 months of age | 0 (0) | 0 (0) | 0 |
| 3 to 12 months of age | 0 (0) | 0 (0) | 0 |
| Lower respiratory tract infection (includes pneumonia, bronchiolitis) | | | |
| 0 to <=28 days | 7 (5.6) | 7 (100) | 0.3 (0.21 to 0.45) |
| 29 days to 3 months of age | 23 (19) | 22 (96) | 0.25 (0.17 to 0.37) |
| 3 to 12 months of age | 94 (76) | 92 (98) | 0.23 (0.2 to 0.27) |
| Meconium aspiration syndrome | | | |
| 0 to <=28 days | 26 (100) | 25 (96) | 0.41 (0.36 to 0.47) |
| 29 days to 3 months of age | 0 (0) | 0 (0) | 0 |
| 3 to 12 months of age | 0 (0) | 0 (0) | 0 |
| Perinatal asphyxia / Hypoxic-ischemic encephalopathy | | | |
| 0 to <=28 days | 23 (100) | 20 (87) | 0.35 (0.27 to 0.45) |
| 29 days to 3 months of age | 0 (0) | 0 (0) | 0 |
| 3 to 12 months of age | 0 (0) | 0 (0) | 0 |
| Sepsis / Serious bacterial infection (includes meningitis)^b^ | | | |
| 0 to <=28 days | 31 (86) | 30 (97) | 0.25 (0.2 to 0.33) |
| 29 days to 3 months of age | 1 (2.8) | 1 (100) | 1 (0.61 to 1.65) |
| 3 to 12 months of age | 4 (11) | 4 (100) | 0.26 (0.13 to 0.52) |
| ^a^ Percent of admissions within the listed subgroup (row)  ^b^ Includes clinically suspected sepsis or serious bacterial infection. | | | |
